# Interdisciplinarity for social justice enterprise: intersecting education, industry and community arts perspectives

**DOI:** 10.1007/s13384-022-00516-5

**Published:** 2022-03-29

**Authors:** Kit Wise, Abbey MacDonald, Marnie Badham, Natalie Brown, Scott Rankin

**Affiliations:** 1 Royal Melbourne Institute of Technology, Melbourne, VIC Australia; 2 College of Art, Law and Education, University of Tasmania, Launceston, TAS Australia; 3 Peter Underwood Centre, University of Tasmania, Hobart, TAS Australia; 4 Big hART, Burnie, TAS Australia

**Keywords:** Interdisciplinarity, COVID-19, Collaboration, Social justice, Education, Arts, Culture

## Abstract

The role of interdisciplinarity in achieving authentic and transformative learning outcomes is both contested and complex. At the same time, traditional disciplinary ways of being, doing and knowing have been further tested by the impact of COVID-19 on students, schools and communities. In Tasmania, already experiencing amongst the lowest levels of educational attainment in Australia, the educational implications of COVID-19 have been polarising. Preliminary reports have employed interdisciplinary perspectives to understand how the situation is unfolding. Extremes of privilege and poverty have intensified, with accentuated disadvantage experienced by already vulnerable groups, whilst ingenuity, adaptability and innovation have flourished elsewhere. The spectrum and range of this polarisation yield compelling evidence for the inadequate address of complex societal problems through singular disciplines or institutions. This article explores storied data generated from the intersections of interdisciplinary strategy enacted across three settings: education, creative industries and community-based arts practice. The data derive from two Tasmanian case studies where interdisciplinary collaboration between the education sector, creative industries and community is well established. In subsequent discussion, the multidisciplinary authorship team make and offer meaning from participatory lived experiences of pursuing social justice outcomes prior to and during the COVID-19 pandemic. From this, we posit how lived experiences of interdisciplinarity impact social justice enterprise in times of increasingly complex socio-economic challenge. In addressing these concerns, we elucidate the role interdisciplinarity plays in both enabling and inhibiting social justice imperatives shared across education, creative industry and community-based arts practice immediately prior to and during a global pandemic. In so doing, we elicit the ways interdisciplinary practices, partnerships and priorities recalibrate in response to global challenges.

## Interdisciplinarity: context and opportunity in lutruwita/Tasmania^1^

lutruwita/Tasmania is the island state of Australia, home to beautiful landscapes of rugged beach and deep forest with unique flora and fauna. This delicate ecosystem also boasts one of the most culturally vibrant creative communities in Australia exemplified by a world-leading museum, exhibitions, festivals and projects initiated by the Museum of Old and New Art (MONA) as well as other island-based creative industries. However, there are also significant socio-economic challenges, including the lowest level of educational attainment in Australia (Stratford et al., 2016). Rowan and Ramsay (2018) describe how “the scale of educational inequality within Tasmania, and between Tasmania and the rest of Australia, has largely been underestimated and its causes misunderstood” (p. 279). This article describes lutruwita/Tasmania as a distinctive place and frame of reference within which we explore the ways interdisciplinary educational projects negotiate wicked problems (Rittel & Webber, 1973) in dynamic social justice enterprise contexts. In this article, we work between Rittel and Webber (1973) and Conklin (2001) conceptualisations of wicked problems, which comprise an evolving set of interlocking issues, constraints and possibilities.

The inherently interdisciplinary ‘STEM’ cluster—science, technology, engineering and mathematics—has been identified as a national education priority in Australia (MacDonald et al., 2019). However, these disciplines are not necessarily aligned; they carry inherently interdisciplinary challenges. The National Science Foundation in the United States of America originally coined STEM in the 1990s for a specific strategic purpose; to recognise the importance of the four STEM disciplines in the economy and as a basis for innovation (Johnson et al., 2015). Rather than being defined collectively for cross-curriculum, educational purposes, combining these four disciplines into the STEM acronym has contributed to the creation of both alliances and hierarchies in relation to other disciplinary clusters such as HASS—Humanities, Arts and Social Sciences. The perceived dominance of the STEM disciplines can be traced earlier than the 1990s and has significantly influenced policy setting and educational trends since the 1960s (Mohr-Schroeder et al., 2015). Teachers have shown tremendous ingenuity in their response to prioritised financial investment in the STEM education agenda globally (UNESCO, 2020). Further, this has been achieved in the midst of escalating global uncertainty caused by a changing climate (Cole & Somerville, 2017; Nairn, 2019) and the COVID-19 pandemic (Coleman et al., 2020); all of which contribute to the exacerbation of perennial educational problems in Australia, such as digital divide and educational inequality (Ng & Renshaw, 2020).

In the Australian context where this study is positioned, there is currently an explicit national drive for increased STEM graduates entering the workforce (particularly females), as evidenced by the recent Federal Government scheme *Jobs Ready Graduates Package* (2020). Pathways into STEM, and higher education more broadly, is a key social justice issue in lutruwita/Tasmania. To assist with this agenda, teachers are encouraged to engage learners with concepts through real-world, authentic projects and challenges. In Tasmania, agriculture is a prominent focus for the integration of science, technology and engineering. With almost one third of the island’s land area (~ 68,000 square kilometres) currently being consigned to agriculture, this interdisciplinary cluster is recognised as being critical for the prosperity of lutruwita/Tasmania (MacDonald et al., 2019). Consequently, the Tasmanian Government supported the creation of new subjects for Years 11 and 12 as part of the Tasmanian Agricultural Education Framework (2016). This recognition of the educative, sociocultural and economic potential of lutruwita/Tasmania’s natural assets was further emphasised by the Tasmanian Government’s investment of $4.9 million over a period of 4 years to provide 10 new teaching staff to support specialist farm schools.

## Surviving and thriving: arts, culture and education

On the heels of Australia’s 2019 summer of ravaging bushfires, 2020 ushered in the COVID-19 global pandemic. The impacts of these successive crises cast in stark relief how different tiers of government perceived the role and value of the arts, culture and education sectors’ economic and social contribution (Banks & O’Connor, 2021; Pennington & Eltham, 2021). In Australia, adjustments to public health policy settings occurred across all levels of government in response to COVID-19. These changed policy settings exacerbated the prior and enduring difficulties many people face in being able to access, practice, participate in and contribute to arts, culture and education (Selkrig et al., 2020). In response, arts, culture and education practitioners demonstrated considerable agility in responding rapidly and effectively, ‘pivoting’ practice, programmes and operation from physical to digital contexts (Hargreaves & Fullan, 2020).

Many schools, cultural institutions and social change organisations in Australia moved to online delivery of services during the height of the COVID-19 lockdowns in 2020. Again, this intensified existing inequities pertaining to digital access, further impacting people already socially and financially disadvantaged. Only 68% of Australian children aged 5 to 14 living in disadvantaged communities have internet access at home, compared to 91% of students living in advantaged communities (Graham & Sahlberg, 2020). These communities navigated immediate problems of retaining work, as well as providing alternative means for community access and engagement during COVID-19.

When social and economic crises converge, as they did in 2020, known thresholds of disadvantage, privilege, innovation and redundancy recalibrate. However, the wickedness inherent to problems that arise from these shifting thresholds create opportunities for critical reimagining, innovation and co-designed solutions. These are spaces where the arts and sciences converge on common ground for common good (Burnard et al., 2021). The new dialogues generated may enhance the political and social consciousness of those directly impacted, which in turn enables greater expression of solidarity and the transformation of collective identity (Campana, 2011). Indeed, in the face of ongoing social and economic challenges globally, the Organisation for Economic Co-operation and Development (OECD) predicts that education and cultural enrichment will take place increasingly through more diverse, flexible and collaborative arrangements, with digital technology a key driver (OECD, 2020).

Collaborators’ disciplines of origin are an important consideration when designing interdisciplinary encounters, outcomes and enterprise. Biases, inhibitors and enablers exist in the spaces between disciplines. Investigation of these prompts thinking about the ‘home’ disciplines for individual educators; their prior knowledge-making paradigms and associated learning and teaching practices (Carey & Matlay, 2010). Through interdisciplinarity, teachers, students and collaborators develop *reflexive vigilance*. This reflexivity exists as a productive tension: making space for other disciplinary ways of knowing, doing and being (Warren et al., 2020) concurrent with, rather than undermining or compromising, one’s own disciplinary skills, strengths and preferences. Championing a reflective, reflexive and relational approach to interdisciplinarity may enable practitioners to reconsider their own discipline-centric knowledge-making processes (Marshall, 2014).

Interdisciplinary aspiration in education contexts calls for relational, brave and generative spaces for meaning making. The distinctiveness and potential of interdisciplinarity may be better understood by focussing on productive tensions, reflexive vigilance and fostering of connections between disciplines as well as contextual settings (MacDonald et al., 2019). In this article, by rendering the enablers and inhibitors of interdisciplinary knowledge, skills and practices in specific case studies, the authorship team consider how interdisciplinarity in action becomes integral to equipping young people for uncertain futures.

## Authorship assemblage: mapping connections

The authorship team bring a breadth of disciplinary and practice expertise across a range of creative industries, education and community contexts; itself an interdisciplinary, praxis-based assemblage (MacDonald et al., 2021). They also have extensive experience of collaboration through prior publications and shared projects. The assemblage of meaning generated from the identified datasets draws on these past and ongoing experiences of collaboration in research and community arts initiatives, primarily in lutruwita/Tasmania, Australia. To map the existing and potential connections that underpin this analysis, we briefly note the individual professional contexts of the authorship teams. Kit Wise is an artist and Dean of the School of Art at RMIT University and has engaged in an advisory capacity with creative arts schools on course design and interdisciplinarity in Australia and overseas, including Singapore, New Zealand, Canada and Australia. Abbey MacDonald is an artist, researcher and teacher working in Higher Education (Arts teacher education) at the University of Tasmania. Marnie Badham has a 25-year history of art and social justice in Australia and Canada, with her research sitting at the intersection of socially engaged art, participatory research methodologies and the politics of cultural measurement. Natalie Brown is the Director of the Peter Underwood Centre at the University of Tasmania and began her career as a teacher of 7–12 Science and Mathematics, in both the North-West and Hobart regions of lutruwita/Tasmania. Scott Rankin is Creative Director and CEO of the renowned arts organisation, BighART. An award-winning author and playwright, Rankin works with over 50 communities in regional, remote and urban Australia.

A collaborative authorship approach supports our assemblage of objective and collective “considerations of emergent theory embedded within narratives” (Leggo, 2008, p. 4). We acknowledge that our prior diverse practices and disciplinary experiences, interdisciplinary collaborations, as well as challenges, form a foundation and lexicon for this article (Baguley et al., 2021). This allows different forms and insights to emerge, which may become the focus of and context for further inquiry (MacDonald et al., 2019). Finally, in developing this article, the authorship team collaborated entirely in digital spaces at different times over a period of 1 year.

## Inquiry architecture: comparing pre- and COVID-normal data sites

Through this process, an initial question was defined: *What interdisciplinary tensions and opportunities arise for personnel involved in education, industry and social justice enterprise initiatives*? This question led to the selection and comparison of two discreet Tasmanian data sites, one generated pre-COVID (2019) and one during COVID-normal (2021): *24 Carrot Gardens* (24CG, 2019) and the *Kelp Pollen Rain Soil* (KPRS, 2021). Both community arts-based projects are outlined in detail below. A second question was then defined: *What key issues/priorities emerge from between these data sites*? Examination of these questions occurred through a shared, collaborative written document, generating a thematic analysis that unfolded in relation to a final question: *In what ways does interdisciplinarity contribute to social change outcomes in education, industry and community arts settings*? This article presents responses to these three questions.

The data sources consist of five semi-structured interview transcripts conducted with five participants, including convenors and producers of the two interdisciplinary social enterprise initiatives identified. These community arts-based projects were selected as exemplars of mature, sustained arts practices with proven experience in interdisciplinary engagement. Each interview explored: *What interdisciplinary tensions and opportunities arise for personnel involved in education, industry and social justice enterprise initiatives*? From these semi-structured interviews, textual data sites were generated for both 24CG and KPRS. Articulating initial points of interconnection and opportune spaces between pre-COVID and COVID-normal supported a systematic approach for considering large amounts of textual information in an unobtrusive way (Mayring, 2004). Our process involved analysis of patterns and frequency of words and structures of connective and communicative discourse (Hsieh & Shannon, 2005). The situational voicing of participants from the data sites are *italicised* throughout.

In this article, we practise reflexive vigilance through attending to and sharing curiosity, risk taking and working with precarity. We present shared meaning, made between disciplinary data perspectives, with an awareness of messiness (Schöch, 2013). In so doing, we demonstrate an understanding of the fluid circumstances in which this article makes relational meaning between data sets that are disparate in time, circumstances and location. The following provides a summary account of the data sites addressed by this article.

## Data collection and findings: data site 1 (pre-COVID-19): 24 Carrot Gardens

The state-wide programme consists of a network of 15 schools, including a new community garden in Bridgewater, that deliver the Stephanie Alexander STEM-based curriculum (Badham et al., 2021). This curriculum provides a “seed to table experience, offering primary school children between the ages of 8 and 12 years, the opportunity to plant, nurture, harvest, prepare, and share fresh, nutritious, and seasonal food” (Block et al., 2012, p. 420). Initiated by Kirsha Kaechele in 2014, curator at the Museum of Old and New Art (Mona) in Hobart, the original vision of 24 Carrot Gardens (24CG) was to *‘*sow seeds of lifelong learning’ in relation to health and wellbeing across school communities. What has eventuated over time is a complex relationship between the creative goals of an art museum and artists with a site-based learning programme across environmental education and science, stimulating strong social networks and a growing foundation of local philanthropic investment. Driven by values of equity and justice for young people growing up in generational poverty, 24CG is a rich and porous example of interdisciplinary education bringing together artistic, environmental and economic aims.

24CG brought together a wide range of stakeholders including teachers, students, parents, local businesses, artists and community arts organisations in the creation of these arts-infused gardening projects, forming mutually sustaining networks. These ways of working are recognised as part of the larger artistic movement over the last four decades of socially engaged art, in which artists activate forms of collaborative practice to identify and affect local social issues (Bishop, 2005). They typically use a range of educational and aesthetic strategies to grow strong intersectoral partnerships.

In our 2019 case study, we spoke to five members of the interdisciplinary 24CG team. Founder Kirsha’s vision was animated by complementary expertise: Sarah Proud, Community Programs Manager and two School Project Managers: Tamas Oszvald (community art, performance, permaculture) and Reuben Parker-Greer (horticulture, education). Kirsha described this coming together:

...taking these really diverse elements from so-called unrelated realities… And then the problem in one world is the solution in another. All these parts can fit together really well but you have to actually take in the big picture, not just look at the one part. (Kirsha, 2019)

We also visited a school to view the garden site and meet with Tristan Bunker, Community and Partnership Coordinator, whose role was to support the school programme to connect to other community groups like Men’s Shed or the Neighbourhood House. Tristan suggested that creating these relationships across difference is *what makes it sustainable and how each school dynamic is different including the students, specialists and teachers*. By connecting different stakeholders and sharing resources helps to achieve self-sustaining economic systems. At the same time, learning from differences between systems allows local, place-based strengths to emerge as well as the innovation in comparable locations. Discourse analysis of this suite of pre-COVID-19 interview data identified the following themes.

### Art and engagement

Artists have the capacity to identify connections across differences and make them visible by growing shared interests through creative and community engagement. Badham (2019) outline how the artistic contributions of 24CG are understood in three ways Firstly, there is an acknowledgement of the intrinsic artistic value of the project, described in relation to the glamorous, global reputation of esteemed museum and associated festivals as ‘Mona-esque’ or ‘gold factor’. Secondly, professional artists contribute to 24CG through a series of connected creative projects including mediums such as gold-plated ceramics. Further to this, each 24CG project includes a Spring Carnival, which brings all the schools, staff and students together to celebrate the programme through spectacular performance festivities. Kirsha recognises the fine balance required between the aesthetic and social interests in a project, a well-rehearsed debate in socially engaged arts (Bishop, 2005; Kester, 2006). She wanted the students to create an environment so beautiful, they would be proud to be in it and proud to share it. She has a concern that *aesthetics are often downplayed in social project*s, arguing that beauty is critical. The artistic and creative value of 24CG is not an ‘add-on’ to a school garden project. It is the inherent driver that exposes and strengthens the visibility of local social and aesthetic connections and expands the cultural dimensions of public experience.

### Economic investment and outcomes

At the same time, the artistic and creative values embedded in the project are also attractive to the project sponsors. As Kirsha describes: “They’re not just doing it because they’re do-gooders and they want to help these poor kids, they’re doing this—this is gorgeous, it’s cool, it’s cutting edge, it’s stylish and they want to be a part of it” (Badham et al., 2021, p. 115). A wider distribution of the arts and different purposes has been documented as demanding partnerships with other sectors that result in new forms of decentralised patronage (Hawkins, 1993). The economic value realised through 24CG took form through a unique donor programme and its ability to leverage government funding and local resources. The programme is a social enterprise, seeking investment in the long-term development of students as cultural citizens whilst also providing training and opportunities to contribute to the agricultural and artisan industry. The collaboration developed between philanthropy, private industry and state government*,* focussed on local priorities and the founders’ interests is an unusual but strong economic model*.*

### Place-based environmental learning

Engaging students with an array of place-based sensory experiences that extend what may be available in classrooms can help cultivate diversified appreciation of nature’s aesthetics and the reception of knowledge. Place-based environmental learning encounters leverage smell, touch, taste and experience of phenomena in varied environments. As such, place-based environmental education experiences are often relational, active and highly affective. Attributes of affect arise in the embodied experiencing-*with*, where sensorial thinking and feeling entangle between place and person in context. This creative production of difference in place can encourage affective transitions in thinking, feeling and being (Braidotti & Bignall, 2018). This topic and its potential for ecological activism was implicit (rather than explicit) in 24CG, potentially due to the political complexities surrounding environmental debates in lutruwita/Tasmania.

None of the public schools actually go there officially, because that goes to politics actually, so...It’s part of the Stephanie Alexander [program], you talk about the waste, how you look after your environment. So, it’s all there, but it’s not sophisticatedly, politically felt. (Tamas, 2019)

Further to this, Tristan notes:

I think because we’re focussing on horticulture and permaculture concepts, we talk about composting at a very minimum. We talk about water resource sustainability. Admittedly I need to do that more. (Tristan, 2019)

Tamas and Tristan speak to challenges and tensions that Sobel (2004) has described as the need for an education movement that not only brings education back into the neighbourhood, but also connects students with artistic and environmental mentors and local business. Socially engaged arts programmes such as 24CG function in the liminal spaces ‘between’ disciplines, community and organisations. Adapting Deleuze and Guattari’s (1988) liminal space as the ‘in-between and around’ disciplinary and organisational edges, where interpretations can flow with dynamic momentum (MacDonald & Moss, 2015), teachers, students, artists and investors are able to make and take risks; to reimagine how curriculum stimulates local social change with impacts on health and wellbeing. Liao (2016) has argued the transformative potential of the spaces between disciplines. 24CG’s integrated and generative approach locates teaching and learning transformation in these relational ‘inter-spaces’ between disciplines.

## Data site 2 (COVID-normal): kelp pollen rain soil

Initiated by a leading Australian arts and social change organisation, BighART on the North-West Tasmanian coast, the project builds partnerships that enable communities to work meaningfully from and for the unique strengths, qualities and challenges of place. BighART uses the ‘canary in the coalmine’ as an overarching metaphoric tool for coming to terms with living positively and hopefully with advanced warning of and/or being in danger.

Kelp pollen rain soil (KPRS) is an interdisciplinary social change project designed to foster resilience, community belonging and to deliver identity strengthening activities for young people from rural communities. To achieve this, the project takes an intra-state, place-based approach, connecting four diverse communities across the island, to deliver four sets of workshops, which generated four linked, one-day arts-driven festival events each based on one significant, place-based Tasmanian asset—kelp, pollen, rain and soil. These four themes have similarities ‘in place,’ and have social, ecological and economic virtues, values and concerns (Rousell, 2020). These four remarkable natural assets are in equal parts abundant and at risk in lutruwita/Tasmania.

At the time of writing this article, the KPRS project concept had been established by BighART, University of Tasmania and RMIT University, with funding secured from the Tasmanian Community Fund for its delivery during 2021. Data generated for this case study centred around articulating the perspectives of key project managers from BighART involved in the conceptualisation, design and delivery of the KPRS project, Angela Prior and Rachel Small. Developed specifically for delivery during/post-COVID in lutruwita/Tasmania, Angela explains how holding the tension of interdisciplinary process and products for arts and social change outcomes *is at the core of everything we [BighART] do.*

Key priorities such as attributes of place, young people and connection feature prominently in the data generated from the COVID-normal interview transcripts and are summarised within the following themes.

### Engaging young people in local industry

The KPRS themes share relationships ‘in place’ as well as having significant social, ecological and economic value. Rachel describes how *for young women in regional areas where agriculture is king… certain industries dominate everything.* School engagement and STEM outreach programmes designed to foster young women’s interest in agriculture and its associated physical sciences have risen to meet the dominance of agricultural industries that Rachel speaks of. Some of these include *Attracting Girls to SET* (Science, Engineering and Technology), linked to KPRS as a parallel project, which sought to “initiate an educational cultural change by diversifying the concept of engineering offered to girls in middle and secondary schools in the state of Tasmania” (Little & de la Barra, 2009, p. 440). Angela talks about the potential of *our culture of export…looking at what it is for young people to be making and exporting content about these* [natural assets] *and connecting and having and driving conversations through these about their future.* For such initiatives, urban, rural and suburban areas must embrace a radical entwinement of informal and formal learning, out-of-school experiences and activities designed around a place-based mission (Vander Ark, 2016). The KPRS project provides a vehicle and support through partnerships to enable the emergence of such an approach.

Angela talks about the ways *socially engaged projects always cross over multiple disciplines* and refers to a further Tasmanian parallel to KPRS as an example. The *Midlands Restoration Project* was a series of ‘Species Hotels’ (Hall & Sutczak, 2019) realised from a collaborative inquiry into the endangered fauna of the Central Midlands habitat. The project synergises with the multiple stakeholder and place-based aspects of KPRS’s social design through collaboration between University of Tasmania staff and students, local Tasmanian Aboriginal community, industries, community and the school of Campbell Town in the Northern Midlands of lutruwita/Tasmania. The project explored and applied “interdisciplinary approaches to regional environmental challenges through development of public art works” (MacDonald et al., 2020, p. 231). *The challenge is in making spaces where industry, arts, education and scientists are all working towards the same outcome and we can have those conversations comfortably* [Angela]*.* This resonates powerfully with the tension of transformational experiences (Meyer & Land, 2005) where the process must sufficiently challenge students’ perception of what is involved, or what is a good solution through play, testing, risk taking, reflection and discussion.

### Place, environment and anxiety

Place-Based Education (PBE) provides a means for teachers and learners to co-construct and shape inquiries in consideration of individual interest and community needs (Coughlin & Kirch, 2010). When Angela talks about how *young people now particularly in Tasmania are highlighting that the environment, inequality, mental health and wellness are important to them,* there is a sense of the attributes, entanglements and potential that PBE can yield and foster in relation to KPRS assets. Through the KPRS, each community will explore one of the natural asset themes; whichever is identified as significant, unique and at risk. Angela explains the importance of such *issues embedded in the social design of these four themes of kelp, pollen, rain, soil, where these assets within Tasmania are becoming vulnerable;* engaging with interdisciplinary perspectives through creative practice-based workshops; the outcomes of which were showcased in four distinct and interconnected site-specific events.

Aspirations to live positively and hopefully, with advanced warning of and/or being in danger permeate this project. These aspirations drive encounters, meaning making and depictions of environment that are never inert; we engage, re-work, appropriate and contest, becoming entangled with the ways our identities are created and disputed in relation to place (Brigham, 2016). KPRS serve as rich natural assets and a ‘canary in the coalmine’ for communities across the island. What emerges is a multidisciplinary metaphoric touchstone for reflective and reflexive tension holding. Rachel and Angela both attest to the convergence of disciplinary ways of knowing, doing and being as significant to the KPRS mission. This is apparent in Angela’s description of *the challenge of knowing how much space to give each discipline and then being able to hold that space in tension with achieving the creative social change outcome that everybody is working towards*. KPRS creates sites and multiple contexts for convergence, where young people are encouraged to utilise a range of interdisciplinary practices and processes to create and leverage productive tension from the spaces between disciplinary ways of knowing, being and doing. It is in and through these that new or alternative relationships with, and storylines about place are created. These are the points for attuning with local, discreet, deep literacies of place.

The metaphor of the ‘canary in the coalmine’ supports development of these literacies in ways that equip young people with tools for navigating interconnections rhizomatically (Deleuze & Guattari, 1988). These approaches actively engage young people in and with the tensions of possibility and peril; where we see narratives emerging that indicate generative perspectives and thinking strategies for manoeuvring through challenges, such as climate change and climate anxiety, together.

### Social justice, change and equality in COVID

Place is not always a driver of thriving reinvention. The acquisition of literacy assets for young people and their families may pose complex challenges. These challenges are often exacerbated by tensions and issues pertaining to access and equity. According to results from limited standardised measurement tools such as NAPLAN, young people on the North-West coast of the state have amongst the lowest levels of literacy—as contained to reading, writing and text comprehension—nationally (Allen et al., 2018; Merga, 2020). These tensions surface in Rachel’s commentary around working with young women and their families during COVID lockdown in lutruwita/Tasmania, who might be categorised as having [according to this narrow definition] *pretty low literacy not only for themselves but a lot of their families,* where *navigating school work became incredibly overwhelming for a lot of them.* It is important to remain vigilant to the complexities of place, and how the global pandemic magnified perennial educational problems in Australia, such as digital divide and educational inequality (Ng & Renshaw, 2020). The KPRS concept is sharply attuned to this, with its social design manifesting from a range of complex interlocking experiences and dilemmas inherent to problems of a wicked nature.

Angela explains how *a lot of young people dropped off* [participating in online connection opportunities] *because their families needed them to work. If they were going to be home, then they needed to be on the farm helping.* Public health lockdown measures implemented during COVID-19 meant that digital innovation became both a significant enabler and inhibitor of people connecting with each other and to place. Some of the young people participating in KPRS during peak COVID-19 lockdowns in lutruwita/Tasmania appear to have found themselves in the thick of a wicked problem. The circumstances that Angela describes allude to complex dilemmas. Some of the young people participating in KPRS clearly grappled with interrelated and sometimes competing demands between digital and physical place. This highlights both the challenges and possibility of digital and place-based pivots, including the implications for how young people access, participate in and contribute to culture.

### Interdisciplinarity for social justice enterprise: themes emerging from the data sites

The data sets generated by the above case studies suggest that the primary educational work required to achieve social justice is an understanding of place and how to best leverage that for maximum learning. Both projects are underpinned by strong social missions to bring about positive change (Witzany, 2005) for the young people and the communities in which they work. The investment of time and energy in place is critical for social change.

**Fig. 1:**
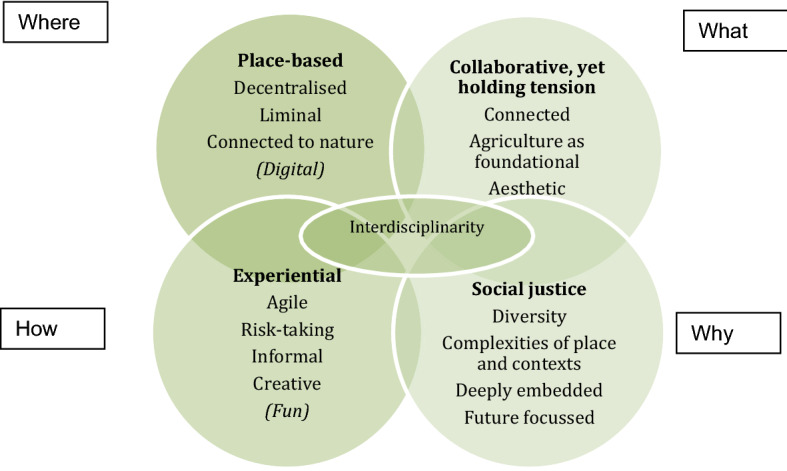
Interdisciplinarity for social justice enterprise

Figure 1 represents the emerging themes through a consideration of programme approach (the why, where, what and how). Through an analysis of the themes from both data sites, four intersecting concerns were identified. The programmes are experiential, place-based, highly collaborative and explicitly address issues of social justice through an understanding of the complexities of place, context and participants. To extend the above focus on place and context, this analysis reflects the recognition in both projects that social change, and indeed social justice is framed as a wicked problem (Rittel & Webber, 1973), founded in a “dynamic social context” (Conklin, 2001, p. 8). The tension reported in the cases, through the interdisciplinary work, are similarly reflected in a broader consideration of wicked problems, complex, uncertain and with multiple competing interests (Head & Alford, 2015). This reflects a real-world application of what has previously been recognised in the educational context (Hawkey et al., 2019), the power of wicked problems to drive interdisciplinary practice, and lead to the generation of collective knowledge.

These four concerns and their intersections can be considered in relation to the second question posed by the investigating team: *What key issues/priorities emerge from between these data sites?* The place-based nature of the projects provide common experiences, linked to the familiar. In so doing, nuance of place is embraced through a decentralisation of decision-making and connection to the natural world. This is where principles of Place-Based Education (PBE) offer a rhizome for connecting people and place through KPRS. Principles of PBE coupled with the ‘canary in the coalmine’ metaphor provide a suite of conceptual meaning making tools for young people and their communities to embrace problems as “generative spaces for possibility” (MacDonald et al., 2018, p. 455), through which they are given opportunity to reimagine deficit storylines and anxiety narratives. Akin to having a language that draws everybody into the project no matter where you are from, this process has the potential to build strong local and state-wide connections through myriad disciplines to rebuild community, economy and identity post-COVID. The events create opportunities to support local businesses, industries and artists, promote conservation values, highlight and celebrate local identity, and strengthen community connection to place.

Qualities of liminality resonate across the projects in ways that mirror the nature of interdisciplinarity. A liminal space is not owned by one entity; its unbounded nature opens up possibilities that are not restricted through conventions of one discipline, or through single ideas or positions (e.g. Shortt, 2015). The power of liminal spaces in an educational context have been promoted by Meyer and Land, as potentially transformative. Allowing exploration of ‘troublesome knowledge’ (Meyer & Land, 2005) in new, different, even playful ways can provide learners with a key to understanding threshold concepts that unlock new learning. In these projects liminal space exists between disciplines, participants and place in ways that invite creativity and co-construction of experiences.

The concept of liminality also represents the inherent complexity in the notion of ‘place’. Whilst it can represent the familiar through mutual experience and context, it cannot be presumed to be common to all participants. This complexity became heightened in the COVID adapted case, where the diversity of capital (manifested in digital inclusion) became central to access. Interviews with Angela and Rachel indicate the increased value and sense of appreciation for pre *and* post-COVID ways of working; and the importance of flexibility and not being bound to particular ways of working. Outcomes they described included having to gain and embrace different disciplinary skills, knowledge and ways of working. These require a rethinking and reimaging of how to hold interdisciplinary process and product in tension with each other. The COVID attuned experience of holding tension feeds directly into the social design and implementation of KPRS. Lockdown forced a change in medium from in person to digital, and this not only enabled new ways and means for connecting, creating and collaborating; it also fostered a renewed sense of appreciation and value of ‘in person’ and ‘in place’.

In both sites, the experiential nature of the projects demanded an agility through flexibility, and the need to adopt both formal and informal processes and structures. The element of risk taking is embraced to heighten creativity. This heightening becomes apparent in granting permission to risk make and take; to generate new and diverse ideas and products (Harris & Carter, 2021). 24CG is bold in embracing ‘fun’, encouraging outward-facing engagement and public celebrations as contributors to the social enterprise. An appeal to a range of philanthropic, government and industry backers is central to the model. This is bolstered through being able to articulate and share the process as well as raising awareness of the underpinning social justice outcomes. Imbuing a community outreach model with principles and possibilities emerging from the ‘canary in the coalmine’ metaphor enables KPRS to focus predominantly on supporting young people and their communities.

The foundational importance of agriculture is evident in both programmes. For 24CG this is as a critical link to the food we eat, for KPRS to reflect the importance of this industry to the local communities. This basic need is held in concert with a strong human connection to the aesthetic. Explicit in 24CG, and reflected through the making of public art in KPRS. Key personnel from BighART describe how kelp, pollen, rain and soil are seminal yet fragile environmental and industrial drivers for Tasmania. During COVID-19 lockdown in lutruwita/Tasmania, Rachel describes how some of the young people she worked with seemed to reinvent and foster new or renewed connections to place; and how this *landing in place and then restarting and rebooting place and values around place has been a really great platform to launch a project like kelp pollen rain soil.* Of significance to KPRS is the wider collaborative interdisciplinary ecologies that are being fostered in lutruwita/Tasmania.

When driven by young people in place and in collaboration with local community and industries, these projects utilise diverse disciplinary skills essential for navigating complex cultural and ecological problems (Rousell, 2020). Communication and community engagement are key drivers of social change (Keller et al., 2015). The adoption of an interdisciplinary approach broadens the modes of communication and sends a powerful message of the value of diverse perspectives.

The following conclusion is articulated as an innovative, interdisciplinary ‘coda’, that draws on theory and practice from science and the creative and performing arts. It holds learnings from the data sites and their analysis in productive tension with alternative voices and practices, supporting new forms of knowledge production that posit an interdisciplinarity in action.

### Entwining education, industry and community arts: conclusion as reflective coda from the field

It is in these spaces and places that young people can begin to see themselves through the lens of ‘yes, and?’

Yes, I am a scientist, and an artist, a thinker, a maker. Yes, I am pushy and a helper, and an inventor. No, I’m not a passive ‘Jobseeker’. I’m my own side hustle, my invention is my self/selves. No, it is not a job for life. It is life.

And this transformative educative moment becomes the multidisciplinary platform of the self-entrepreneurial journey.

This article converges 30 years of practical experience in and evidence from the field to elicit the ways project designs—such as KPRS and 24CG—need to be structured as reflexively interdisciplinary ways of knowing if they are to actually be transformative. To return to the final question raised by the investigators: *In what ways does interdisciplinarity contribute to social change outcomes in education, industry and community arts settings*? Transformative learning outcomes (Liao, 2016) are not about mentors, teachers and artists. It is young people who do the transforming. Being ‘a youth’ is by nature liminal; a syncopating breath between child becoming adult, dependence becoming independence, expansion becoming contraction of growth (Meyer & Land, 2005). Just as play is the work of childhood, young people’s search for self, place and belonging is the work in the teenage years.

The trial and error of this identity work is not linear, it is inherently interdisciplinary. It is a passage where emergent identities shapeshift playfully and rambunctiously. When young people are well supported in this self-searching, if one set of choices fails, a healthy rhizomic web (Deleuze & Guattari, 1988; Witzany, 2005) of other self-entrepreneuring experiments will hold a young person safely, as new shoots and roots pop up through the rich loamy soil of the teenage trial and error. Our interdisciplinarity projects must feed this soil, and seed resilience and self-innovation. We posit that young people can and do flourish within this kind of flow.

If, however, our projects impose simpler mono-disciplinary approaches that risk ring-fencing young people into convenient, homogenous pathways for tertiary or labour markets, we rob young people of the curious, complex layered experiences they crave. The young person who recoils from this ring-fencing through classroom antics may be signalling more than just disruptive behaviour; they may be pursuing a kind of self-preservation from our clumsy prescriptiveness. Perhaps their behaviour wraps around a form of self-structured interdisciplinary inquiry, forging pathways through ‘darker’ expressions of self (Hunter & MacDonald, 2017), in public space campuses on skateboards, through Picasso-esque tags rendered in spray paint, in their body markings, dare and dark play (Cropley, 2010).

Projects like KPRS and 24CG are better seen as invitations into multi-layered systems of being and discovering, rather than welfare or education re-engagement programmes. They provide opportunities for young people to cross paths with virtuosic artists, community builders, scientists and producers, who are ‘already doing’ (Kester, 2006); pursuing new disciplines and ideas in mutuality, poetics, localism, trust, art, ecology and generational learning in places where paths cross (Rousell, 2020). These projects do not target young people in conventional ways; rather, they seek to be discoverable by young people. They are de-institutionalised and holding the door open to strange new worlds and disciplines, inviting young people to opt in.

In the liminal spaces of education, industry and community art projects such as 24CG and KPRS, interdisciplinarity is not service provision, deficit or welfare focussed. It is rigorous art/invention/science making (Burnard et al., 2021). Within these projects, an invitation to fly blind is offered and accepted, with understanding of being seen and held with space created for failure—the compost for growth. Projects such as 24CG and KPRS beckon young people into a set of contradictions and tensions, where curriculum is emergent and does not fit together neatly. You cannot get signed off by being wrote or woke. Achievement is dramaturgical (Benford & Hunt, 1992), with process and content woven together like wrestling eels, examinable through your local community’s wonder and applause. The assessment task is in the interactive adventure, and the gold star is local pride. These kinds of projects are the whetting stones on which young identities—dulled by a lava flow lethargy of educational mediocrity—become sharpened.

How does interdisciplinarity contribute to social change outcomes in education, industry and community arts settings?

Youth focussed projects that can be easily written into government grants or tenders have the potential to tend towards harm in pursuit of easy deliverables (Laudel, 2006). The best educative place-based projects for young people use multi-layered approaches that entwine multiple stakeholders who converge in their support of strengths-based approaches. They are not deficit focussed, with welfare objectives; they are invitations into complexity (Head & Alford, 2015). They assume success rather than failure and demand mutuality, linked to real-world localised experiences. Projects of the ilk explored in this article resist any notion of educational ghettos being set up to babysit under-achievers. Instead, they are configured around the experiential identity work young people are already doing, which is naturally, instinctively and inherently interdisciplinary (Harris & De Bruin, 2018).

With a social justice imperative, the projects examined in this article are driven by the recognition of the need for diversity of experiences and contexts for the young people participating in them. Young people will continue to challenge the porosity of liminal space between disciplines. Is skateboarding for instance, a pastime, repetitive sport, or is it the kinetic analysis of urban architecture through the sensory perceptions of balance, flow and feet? Are these young people wasting time or concurrently building resilience and practising mental health first aid? These are the kinds of questions, possibilities and binaries we make and break at the interdisciplinary junctures (MacDonald et al., 2020) mapped across the place-based projects examined and discussed in this article.

Working towards this end goal is framed as needing to be future-focussed and supporting the development of deeply embedded learning experiences that transcend politics and build confidence and transferrable skills (Hawkey et al., 2019). Navigating the tensions of all players—the participants, their communities, supporters—with abundant ideas, values, experiences and expectations, is at the heart of these projects, their social justice outcomes and probable prototypes into the future. The primacy of collaboration and communication within and across these areas of tension are clear, as is the importance of embracing the broader communities in which this work is being done.

Post-COVID normality presents rare opportunities and circumstances for delivering these transformative hybrid projects. Government ears are slightly less blocked and risk averse at present. And so now is the time for our approach to be radical, deft, smart and calibrated around systems of support for vulnerable young people. To aspire towards justice, educative projects must be more attuned to ‘catch up,’ at a time when those doing well are doing better, and those who are not risk the negative effects of stasis—a kind of policy inertia, flight or freeze. Right now, what disadvantaged young people need is rare advantage, and this rare advantage must be the target of our interdisciplinary project designs, funding and sophisticated action.

## Footnotes

1 Originally named Van Diemen’s Land in 1642 by European explorers, lutruwita is the palawa kani (‘*Tasmanian Aborigines speak’*) name for Tasmania. An island with complex post-invasion history, lutruwita / Tasmania’s truths are surfacing and being re/written by contemporary cultures of the island’s First Nations Peoples. In acknowledgment of the complex histories of place and diverse disciplinary settings out of which this articles’ storylines emerge, we practice dual naming of place in this article. Further information in respect to First Nations place naming process and practices in lutruwita/Tasmania can be found in our reference list (see Tasmanian Aboriginal Centre, 2021; Rimmer & Sainty, 2020).
